# A phenomenological investigation of patients’ experiences during direct observation in residency: busting the myth of the fly on the wall

**DOI:** 10.1007/s10459-021-10044-z

**Published:** 2021-03-25

**Authors:** Chris B. T. Rietmeijer, Mark Deves, Suzanne C. M. van Esch, Henriëtte E. van der Horst, Annette H. Blankenstein, Mario Veen, Fedde Scheele, Pim W. Teunissen

**Affiliations:** 1 Department of General Practice/Family Medicine, Amsterdam University Medical Centers, location VUmc, De Boelelaan 1109, 1081 HV Amsterdam, The Netherlands; 2 Department of General Practice/Family Medicine, Amsterdam University Medical Centers, Location AMC, Meibergdreef 9, 1105 AZ Amsterdam, The Netherlands; 3 Department of General Practice, Erasmus Medical Center, Dr. Molewaterplein 40, 3015 GD Rtterdam, The Netherlands; 4 Amsterdam University Medical Centers, School of Medical Sciences, Athena Institute for Transdisciplinary Research, VU University, location VUmc, Amsterdam, The Netherlands; 5 School of Health Professions Education, Maastricht University, Universiteitssingel 60, 6229 ER Maastricht, The Netherlands; 6 Heemraadschapslaan 33, 1181 TZ Amstelveen, The Netherlands

**Keywords:** Patients' experience, Phenomenology, Direct observation, Participative direct observation, Feedback, Assessment, Patient wellbeing, Patient centeredness, Post-graduate medical education, Residency

## Abstract

Direct observation (DO) of residents by supervisors is a highly recommended educational tool in postgraduate medical education, yet its uptake is poor. Residents and supervisors report various reasons for not engaging in DO. Some of these relate to their interaction with patients during DO. We do not know the patient perspectives on these interactions, nor, more broadly, what it is like to be a patient in a DO situation. Understanding the patient perspective may lead to a more complete understanding of the dynamics in DO situations, which may benefit patient wellbeing and improve the use of DO as an educational tool. We conducted a phenomenological interview study to investigate the experience of being a patient in a DO situation. Our analysis included multiple rounds of coding and identifying themes, and a final phase of phenomenological reduction to arrive at the essential elements of the experience. Constant reflexivity was at the heart of this process. Our results provide a new perspective on the role of the supervisor in DO situations. Patients were willing to address the resident, but sought moments of contact with, and some participation by, the supervisor. Consequently, conceptions of DO in which the supervisor thinks she is a fly on the wall rather than a part of the interaction, should be critically reviewed. To that end, we propose the concept of *participative direct observation* in workplace learning, which also acknowledges the observer’s role as participant. Embracing this concept may benefit both patients’ wellbeing and residents’ learning.

## Introduction

In post-graduate medical education, residents see patients independently, under nearby supervision. The highest level of this supervision is direct observation (DO), which is indispensable for purposes of feedback and assessment (Kilminster et al., 2007; Kogan et al., 2017). In this paper, DO refers to any situation in which the supervisor is physically present, observing the resident working with the patient (Rietmeijer et al., 2018). Notwithstanding its importance, DO has proven difficult to implement in medical education, including postgraduate education. Residents and supervisors report various reasons for not engaging in DO, mostly related to fear of assessment, feelings of mistrust, and expectations concerning autonomy and efficiency (Kogan et al., 2017, Rietmeijer et al., 2018, Rietmeijer et al. 2021, Watling et al., 2016, LaDonna et al., 2017, Cheung et al., 2019, Pelgrim et al., 2012).

Difficulties in interacting with patients form yet another barrier for engaging in DO. Residents may fear that patients see DO as a sign of lack of competence. Also, residents report that they behave “unnaturally” towards patients when an observer is present. Residents may also fear that overt critique by the supervisor can cause patients to lose trust in them. Furthermore, both residents and supervisors experience difficulties in encouraging the patient to address the resident rather than the supervisor. Lastly, both residents and supervisors may feel that DO can be frustrating for patients in terms of being examined twice or having to wait for the supervisor (Rietmeijer et al., 2018; Rietmeijer et al. 2021; LaDonna et al., 2017; Pelgrim et al., 2012).

While we know from all this research that supervisors and residents have various assumptions about what patients think and feel, patients’ actual experiences in DO situations in post-graduate medical education have scarcely been investigated. What we do know comes from a small number of survey studies that examined the effects of DO situations on patient-centered care in residency (Pitts et al., 2015; Starmer et al., 2009); these indicated patients’ general satisfaction with DO situations but yielded no specific information about what it is like to actually be the patient in DO situations.

We can expect that patients’ experiences in DO situations will partly reflect their experiences when participating in health professions education (HPE), more generally. Sharma outlined the literature on patients’ experiences when participating in HPE in a narrative review (Sharma, 2018). Briefly summarized, patients saw participation in HPE as “a means of ‘giving back’ to the medical community”. Patient educators reported among others “raised self-esteem and empowerment […] However, there were concerns around consent, confidentiality, emotional well-being, […] particularly around the sharing of personal or painful health issues” (Sharma, 2018). In a phenomenological interview study of teaching encounters in out-patient clinics, McLachlan and colleagues found that some patients preferred not to be objectified, but instead to be involved in a triadic teaching relationship of mutual benefit (McLachlan et al., 2012). Monrouxe and colleagues found through conversation analysis that patients were often assigned a passive role in bedside teaching, even if they rejected such passivity (Monrouxe et al., 2009). We have, as yet, little or no information about whether, to what extent and how all these findings apply to DO situations.

Summarizing, DO situations are challenging for residents and supervisors. This partly relates to their interactions with patients and their assumptions about how patients experience DO situations. We do not know how patients actually experience these situations. Understanding the patient perspective may lead to a more complete understanding of the dynamics in DO situations, which may benefit patient wellbeing and improve the use of DO as an educational tool. This is in line with Kogan and colleagues’ call in their guidelines on DO of clinical skills in medical education: “How to best create a therapeutic and educational alliance with patients in the context of direct observation requires additional attention” (Kogan, 2017). These considerations led to our research question:

What is it like for patients to have a consultation with a resident while the supervisor is observing the resident?

## Methods

### Context

We performed our interview study in the general practice (GP) residency program at the Amsterdam University Medical Centers, location VUmc. In the first and final year of this three-year program, residents are paired with one, sometimes two, GP supervisor(s) for the whole year. Residents see patients independently, with the supervisor immediately at hand. In Dutch general practice, patients typically have one, in some cases two part-time, familiar general practitioner(s). In GP training practices, patients are used to occasionally being asked to see the resident instead of their familiar GP. Residents do not have permanently assigned ‘own’ patients.

### Phenomenological approach

Since our aim was to investigate the experience of being the patient in a DO situation as openly as possible, we chose a phenomenological approach. The past decade has seen a surge in health professions education (HPE) research that claims a phenomenological approach. These studies differ greatly in how phenomenology, and the methods used, are described, and an animated debate is currently ongoing about what phenomenological research is, and how it should be performed (Bynum & Varpio, 2018; Dowling, 2007; Neubauer et al., 2019; van Manen, 2017; Zahavi, 2019). Based on the ideas of classical phenomenologists, and on contemporary phenomenologists Zahavi and van Manen, we summarize the main features of the phenomenological approach that guided our research (Husserl, 2018; van Manen, 2016; Zahavi, 2018). See Box 1.

**Box 1:** Features of a phenomenological approach, based on Husserl, 2018, Zahavi, 2018, van Manen, 2016

1. Openness to the object of interest, i.e. no pre-fixed theory or categories or even methods; adjust the methods to what the inquiry of this specific object calls for
2. Taking subjective experience into account; it is not about the world (objectivism), nor about the mind (psychologism), but about the interaction between mind and world. It is about how a subject experiences an object. The total of this is the phenomenon. A phenomenon is anything that presents itself to our awareness
3. Interest in the *pre-reflective* experiences of people, i.e. what they experience before they have reflected on it. We are not interested in peoples’ ideas, opinions and feelings per se, but rather in the essential elements of the pre-reflective experience that made these ideas, opinions and feelings at all possible
4. Interest in what individuals’ experiences with the phenomenon have in common
5. Interest in the phenomenon as people experience it in the full context of their daily lives
6. Acknowledgement of the pivotal role of the researcher who is the subject performing the study; investigate this subject as part of the study

We were attracted to this approach because we wanted to know what it is essentially like to be the patient in a DO situation. A phenomenological approach to a persistent problem, such as the continuing issues with direct observation in HPE, may lead to surprising results that could enrich or contradict knowledge from other research approaches. This may lead to clues for new theories and solutions (Varpio & McLeod, 2020).

A prominent feature of phenomenological research, as stated, is the absence of a prefixed method to approach our object of interest (van Manen, 2016). This means that researchers make use of methods that they decide fit a phenomenological approach to their object of research. By being transparent, researchers give insights into how they meet the criteria for rigor. We, therefore, describe our methods in detail below.

### Reflexivity

Since the purpose of a phenomenological analysis is to achieve an understanding of pre-reflective experiences, the researchers have to be aware of their premises and beliefs that may keep them from perceiving deeper layers of experience (van Manen, 2016; p 47). Therefore, before the interviews were performed, CBTR and MD each wrote an essay on their experiences with, and their assumptions about, the phenomenon of being the patient in a DO situation. CBTR is a GP, coach and teacher; MD is a psychologist, coach and teacher. Subsequently, CBTR and MD interviewed each other about these essays. This session was audio-recorded and transcribed. Both CBTR and MD read this transcript and separately wrote memos to capture the experiences they felt were important during DO in medical consultations. As two examples of these, MD thought that a clear role division between resident and supervisor during DO was important for the patient’s ease. CBTR tended to think in terms of learning goal orientation versus performance goal orientation of the resident, and how that affects the interactions between resident, supervisor and patient. There were many other assumptions and beliefs that the researchers came across. All memos were discussed between them and each wrote a summary of all the memos; the summaries were then discussed with FS, PWT, AHB, CBTR and MD, and served as the start of a reflexive diary which was kept throughout the process of analysis and writing to capture our progressive insights. This process helped to challenge our presuppositions explicitly and deliberately, or put them on hold, i.e., “bracket” them (Van Manen, 2016 p 47).

### Data collection: participants and procedure

In five different GP training practices in the western part of the Netherlands, patients who wished to make an appointment were asked if they were willing to see the resident with the supervisor present observing the resident for educational purposes. They were also asked if they would consent to an interview of approximately 20–30 min directly after the consultation. Information about the study was given at the same time. The information was also sent to the patient by e-mail, including an informed consent form, which was signed before the interview started.

This procedure allowed us to include a range of different patients, supervisors, residents and consultations: patients who seldom visit their GP as well as patients that came more often; older and younger patients; men and women; patients after emotional consultations and others not; patients consulting a first-year resident and patients consulting a third-year resident. Exclusion criteria were being aged under 16 and not speaking Dutch or English. In phenomenology, typically, the number of interviews is relatively small while the yield of each interview is relatively high because of the in-depth interviewing and analysis. Eleven interviews were conducted between March and June 2018 by either CBTR or MD. See Table 1 for the characteristics of the participants, and the residents they consulted. All interviews were audio-recorded and transcribed. Transcripts of the interviews were entered in a qualitative software program (Atlas.ti; Scientific Software Development GmbH, Berlin, Germany).

**Table 1:** Characteristics of patients interviewed and residents they consulted; columns 2, 3 and 4 show patients’ self-reported frequency of visits and familiarity with the resident and supervisor ( −  up to +  + +)

Patient: number, gender, age	Patient: average frequency of visits to GP per year	Patient: familiar with supervisor	Patient: familiar with resident	Resident: letter, year of training
1; Female, 44	4	+ + +	+	A; 3rd year
2; Female, 16 (plus mother)	2	+	-	B; 3rd year
3; Male, 29	0–1	+	+ +	A; 3rd year
4; Female, 69	4	+ + +	-	B; 3rd year
5; Male, 61	2	+ +	+	C; 3rd year
6; Male, 63	2	+ +	-	C; 3rd year
7; Female, 78	10	+ + +	+ +	D; 3rd year
8; Male, 67	0–1	+	+	D; 3^rd^ year
9; Male, 68	0–1	+	+	D; 3rd year
10; Female, 23	2	–	–	E; 1st year
11; Female, 28	4	–	–	E; 1st year

### The phenomenological interview

We interviewed the patients immediately after the DO situation, in a separate room at the GP practice. We chose a very open interview format that started with the question: “You have just had a consultation with a GP resident, while the supervisor was present, observing; can you please tell me about this experience?” When patients needed to be encouraged to share their thoughts and feelings, facilitating questions were often about factual issues like “Who sat where?” or “And then what happened?” (Van Manen, 2016 p 131). This was followed by asking how patients experienced particular moments. When particular issues were not addressed spontaneously, the interviewers would bring up issues around DO that are known from previous research among supervisors and residents, as indicated in the first paragraph of the introduction to this paper. The most remarkable outcomes of the interviews were discussed between CBTR and MD in weekly sessions; emerging insights informed subsequent interviews. As an example of this, we had not expected that it would take so much effort to help patients zoom in on how specific aspects of the consultation experience had been to them. This made us go through the consultation they just had even more meticulously, adding more questions about the actual events that took place and how patients themselves had acted.

### Data analysis

We analyzed the transcripts combining van Manen’s three-step approach (see below) with his focus on four ‘existential’ aspects of experience, i.e. lived body (corporeality), lived space (spatiality), lived time (temporality), and lived human relation (relationality) (van Manen, 2016).

Reading the transcripts while paying special attention to these existential aspects of experience helped us bring this experience itself to the fore, while bracketing explanations, theories and interpretations. To give some examples of how this worked, focusing on lived body made us extra sensitive to all quotes reflecting, for instance, nervousness, anxiety or discomfort. Lived space helped us see how the position in the room influenced experience. Lived time helped us see how previous experience could play a role. Lived relationship helped us, for instance, see how the familiarity of the patient with either the resident or the supervisor colored the experience. Differentiating these four existential aspects of experience was not an end in itself, but a means to distill the full experience from the transcripts; it was a step in the analysis. Consequently, in the results section, we do not report on these existential aspects. We do report on the essential elements of the experience as a whole.

- *Step 1* CBTR read every transcript several times, then wrote a ‘sententious phrase’ (this is the term van Manen uses for a concise, meaningful phrase) about what the transcript, as a whole, told him about the phenomenon. Subsequently, he wrote a short reflection on this sententious phrase.
- *Step 2* CBTR read the transcript again, paying attention to anything that related to the bodily experience; all these sentences were open coded (e.g.”lived body: the patient prefers to be examined twice above feeling insecure”). This was repeated three times, focusing on lived space, lived time and lived human relation, respectively.
- *Step 3* CBTR read the transcript one more time, open coding every sentence that gave additional information. To conclude, he added reflections that had arisen during this process to his first reflection on the sententious phrase.

After this three-step analysis, CBTR tried to capture themes that had come up in what van Manen calls linguistic transformations: 100 to 200-word descriptions of elements of the experience (e.g. the patient is -or is not- familiar with the supervisor and/or the resident) (Van Manen, 2016 p 92). CBTR sent these sententious phrases, reflections, and codes and the linguistic transformations of each transcript, one at a time, to PWT, SCMvE and MD over the course of a few months. They subsequently first read the transcripts several times, wrote a sententious phrase and reflection for each transcript themselves, added codes, then read and gave a response in writing to CBTR’s analytical output.

During this process, CBTR, PWT, SCMvE and MD held meetings and discussed disagreements until they reached consensus. A codebook was developed, consisting of 187 codes in 24 groups.

Through several rounds of grouping and regrouping codes, reflections and descriptions, we identified essential elements of the experience of being the patient in a DO situation. In the process of identifying such essential elements, we were guided by the question whether the phenomenon would still be the phenomenon without this element (imaginative variation) (van Manen, 2016). As one example to clarify this, we had many codes about eye contact between the patient and the supervisor; this, therefore, seemed an essential element of the experience. However, we argued that if there were no eye contact, the phenomenon of being the patient in a DO situation would still be the same phenomenon. Eye contact was not essential. What was essential, in this example, was the presence of a second, more senior, often familiar, doctor that the patient could relate to and be reassured by, in which eye contact often played a role.

We set out by analyzing transcripts using all of the steps described above, starting with those transcripts that seemed most informative after an initial reading. Once we reached the point when additional transcripts did not add new essential elements (after six transcripts), we moved to repeated and critical reading of the remaining transcripts, checking for additional information and new (more detailed) codes. The last few of our eleven transcripts did not reveal additional information. We, therefore, did not expect that conducting more interviews at this stage, in our setting, would reveal new essential elements. Saturation, however, is not what phenomenological research aims for. As van Manen puts it: “Every phenomenological topic can always be taken up again and explored for dimensions of original meaning and aspects of meaningfulness” (van Manen et al., 2016).

The team met three times and conducted further discussions per e-mail. After this, several versions of the whole paper were commented on by the same researchers as well as by HvdH, AHB, FS and MV.

## Results

We included eleven patients from five different training practices. Table 1 shows their characteristics.

In the DO situations they participated in, patients felt that they received the care that they came for, while simultaneously contributing to a resident’s learning. They saw this as a win–win situation in that the resident could learn from the patient while the patient benefited from two doctors, and “two heads are better than one” (quote from several patients).

Patient 3: “Yes, I find that important (DO of the resident). This is about the health of your friends, your family […]. I think that the resident should be supervised and should be advised how to improve. Happens to me too in my daily work.”

Patient 6: “And then they both checked, made sure they agreed what it is, and then they had a short discussion about what kind of treatment I could receive. Yes, it was fine, yes.”

Most patients stated firmly that, for the above reasons, they had no problem with the observation situation that they participated in. In many interviews, it proved a challenge to persuade patients that, even in the absence of a problem, all their experiences were of interest to us. Only through ‘replaying the film’ quite factually, and through probing details and repeatedly asking how the patient experienced particular moments did we get an insight into patients’ actual experiences.

## The essential elements of the experience of being a patient in a DO situation

As explained in the method section, through several rounds of grouping and regrouping codes, reflections and descriptions, and through the technique of imaginative variation, we identified essential elements of the experience of being the patient in a DO situation.

We present five essential elements, see Box 2. We will elaborate on all these elements in the next paragraphs.

**Box 2:** Essential elements of the experience of being the patient in a DO situation

1. Patients experienced DO situations as a choice. This resonated with their sense of autonomy. All patients were willing to cooperate, but some patients could think of situations in which they would rather not
2. Patients experienced DO situations as two doctors interacting with one another and with them. This resonated with patients’ need for calm and friendly interactions that helped them feel comfortable in the DO situation
3. Patients experienced DO situations as a junior doctor who was observed by a senior doctor. This signaled that the junior doctor was a learner who was less experienced than the senior doctor. The presence of both a junior and a senior doctor resonated with the patients’ need for good care
4. Patients often experienced DO situations as occasions where an unknown, or little known, doctor was observed by the patient’s more familiar GP. This resonated with patients’ needs for relatedness with, and care from, their own GP
5. Patients experienced DO situations as offering them a new role as collaborators in medical education. This resonated with their sense of responsibility for—and for some patients, engagement with—the education of future doctors

1. Patients experienced DO situations as a choice. This resonated with their sense of autonomy. Some patients reported that they would not always agree to participate, or feel comfortable participating, in DO situations. When they visited the doctor with emotional or very personal problems, for instance, they would prefer to talk to their own trusted family doctor, or to just one doctor, be it the resident or the supervisor.

2. Patients experienced DO situations as two doctors interacting with one another and with them. This resonated with patients’ need for calm and friendly interactions that helped them feel comfortable in the DO situation.

Patient* 10:* “Yeah, I felt that the atmosphere between the resident and the supervisor was good […]. I think they have a good working relationship so then it’s not unpleasant for me.”

Also, patients’ own harmonious interaction with the supervisor and the resident was often mentioned as important for their feeling comfortable in the DO situation. Such harmonious interactions were further described as honest, open, attentive and respectful.

Patient 4: “… and yes, she (the resident) was very understanding and patient. I found that kind of her. And she asked if there was anything else.”

Patients experienced a varying division of roles between the resident and the supervisor, and themselves: Typically, the resident would act as the doctor; the supervisor would, silently or less so, observe from the side; the patient would talk to the resident. However, patients described variations on this typical role division, with the supervisor sometimes being more present, sometimes sitting next to the resident. Some patients said they found it important that the intended division of roles was clearly communicated at the beginning of the consultation. Some presumed that they would find it unpleasant if a certain role division were suddenly breached, e.g. by a too dominant supervisor. In such a situation, they would then empathize with the resident and feel uncomfortable themselves. Paradoxically, many examples were given of actual spontaneous yet harmonious changes in the division of the roles: the resident who consulted the supervisor, the patient who addressed the supervisor, or vice versa, and even the supervisor who intervened:

Patient 6: “Well, she said she did not have the result of the ultrasound, so she suggested she’d first contact the hospital, and then she asked her supervisor if he agreed.”

Patient 1: (after becoming emotional): “I think it made them feel more comfortable that the supervisor explained to the resident where my emotions came from. It made me more comfortable, too, that I did not have to explain about this mild depression…”

3. Patients experienced DO situations as a junior doctor who was observed by a senior doctor. Although patients understood fully that the idea of the observation situation was that they should address the resident, they reported many moments of eye and/or verbal contact with the supervisor in his/her role as senior doctor. Some patients indicated clearly that they needed signs of approval of the resident’s approach by the supervisor.

Patient 7: “just looking at him to check if he thinks the same […]; he has years of experience. Would he know of an alternative of some kind?”

Some patients indicated that they expected the supervisor to actively support good care when needed:

Patient 7: “He did not intervene but he listened attentively and I think he would have spoken up had he known a solution for me.”

Patients said they did not mind the resident asking the supervisor for help. On the contrary, some patients said they *expected* residents to discuss with their supervisor whatever they felt insecure about, in the interests of appropriate patient care. One patient said that she expected the supervisor to play an active role in the consultation. She saw him more as a second doctor than as an observer. Without his explicit approval, she would feel insecure.

Patient 11: “Just to make sure: okay there are 2 doctors that say the same thing and I should be fine. But this is only in the situation where there are 2 doctors there. If there is only on doctor there, I would rely on one doctor’s opinion.”

Constructive discussions between supervisor and resident regarding the diagnosis or treatment plan gave patients confidence that they received good care. Some patients experienced moments when they were not part of this discussion; they understood this and did not mind. They assumed, however, that they would become worried about their condition if the resident and supervisor were to openly disagree.

Patient 11: “For example, the junior doctor is doing the examination, then the supervisor interrupts and says: this is not okay, and there is some disagreement between them in front of the patient. That would not be okay, because it would worry the patient.”

If that should occur, some patients thought they would find it important to be included in the conversation and understand what the disagreement was about.

4. Patients experienced DO situations often as occasions where an unknown, or little known, doctor was observed by the patient’s more familiar GP. Patients might seek interaction with the supervisor in his/her role as familiar GP, as we see in these quotes:

Patient 4: “ […] because he (the supervisor) knew my half-brother X who died from colon cancer. I said I had to think of X, so I turned around to him (the supervisor), he had cared for X so well, so… that was briefly between the two of us.”

Patient 8: “ […] Unexpectedly, it became an emotional conversation, so I was pleased that my family doctor was there because he knows my history.”

Patients valued these moments of contact with the supervisor. If it did not happen spontaneously, some patients intentionally looked for such moments of contact. Even if the supervisor sat behind the patient, as was sometimes the case, there were moments when the patient would turn around to the supervisor. They realized that they were supposed to focus on the resident but did not see these moments of contact with the supervisor as a problem.

Patient 4: “I think it happens unnoticed sometimes […] and afterwards I just turned to her (the resident) again.”

5. Patients experienced DO situations as offering them a new role as collaborator in medical education, as a patient to practice on under DO. This resonated with their sense of responsibility for—and for some patients, engagement with—the education of future doctors.

Although patients expressed modesty concerning their contribution to residents’ learning, this contribution was what motivated them to participate.

Patient 6: “Well, if that leads to better education, yeah well, I am happy to contribute, sure.”

Patients said they did not mind that their cooperation in DO situations entailed some inconveniences, like sometimes being excluded from the conversation between the resident and the supervisor or being physically examined twice.

Some patients indicated that the resident or supervisor had expressed their gratitude for the patients’ cooperation. This added to the patient’s willingness to cooperate.

Patient 6: “She was very glad that I cooperate. Yeah well, then it’s logical that I’m happy to…(help).”

Patients could be enthusiastic about the accomplishments of the resident, and some patients said that they had shown their approval of the resident’s approach to the supervisor, for instance by exchanging a glance.

## Discussion

In order to address persistent problems with DO in residency, including the interaction with patients, we set out to enlarge our understanding of DO situations by investigating patients’ experiences in these situations. Our phenomenological approach helped us trace patients’ various thoughts and feelings back to common essential elements of their experience.

One of our findings relates to the fact that, in the context of GP training, the patient is often familiar with the observing supervisor, as will not be the case in many other HPE settings. Nevertheless, our results may, to some extent, apply to educational settings in other health professions. We will first discuss our results in relation to patient wellbeing then gradually move on to the educational consequences of our findings.

### Patient wellbeing

In line with the broader literature on patient participation in medical education, as described in the introduction, we found that patients said that they experienced DO as a win–win situation: they valued their contribution to education, and they felt that they received extra good care. Our participants rarely experienced adverse effects. This seemed mainly related to the friendly, harmonious atmosphere that they experienced and the possibility of also interacting with the supervisor.

Interestingly, triadic dynamics in DO situations seem different from those in other teaching encounters involving patients. As mentioned in the introduction, patients can be objectified and assigned a passive role in bedside teaching, even if patients rejected such passivity (McLachlan; Monrouxe et al., 2009). Elsey and colleagues, in an ethnographic conversation analysis study, further described how patients are included in, or excluded from, the teaching conversation (Elsey et al., 2017). These findings are insightful in this era of patient-centered care and patient involvement in medical education (Monrouxe et al., 2009). In DO situations, by contrast, our participants, did not experience a lack of their own participation in the triad. Instead, it was the supervisors’ participation or lack of participation that mattered to them. Apparently, in DO situations, patient centeredness is not so much about involving the patient but about all parties being involved, including the supervisor.

### Fly on the wall or elephant in the room?

Current guidelines on DO, such as observing without interrupting the encounter, and sitting in the patient’s peripheral vision, are aimed at creating a DO situation in which the patient and the resident can interact as naturally as possible with the supervisor as the fly on the wall. This should add to the authenticity of the performance that is observed and minimize interference with residents’ autonomy and their relationship with patients (Kogan et al., 2017). These guidelines recognize, however, that patient safety may demand participation by the supervisor. Our findings suggest that patient wellbeing in DO situations is yet another justification for some participation by the observing supervisor. We acknowledge the value of the fly-on-the-wall approach for educational purposes, but identify a risk when supervisors go too far in this, as they sometimes do (Rietmeijer 2018). For instance, when supervisors avoid any connection with the patient and/or the resident, this could create awkwardness and undermine the patient's needs. Patients seemed to need some interaction with the supervisor for two reasons. The first reason may be typical for our context of GP training: to do justice to their relationship with their familiar GP. The second reason possibly applies to all HPE contexts: to verify that the supervisor approved of the approach of the resident. For this last reason, some patients also wanted to see some interaction between the resident and the supervisor. Therefore, supervisors who exaggerate the fly on the wall approach by pretending not to be there risk making themselves the elephant in the room.

This elephant may, to some extent, be the cause of problems that both residents and supervisors encounter in their interaction with patients in DO situations, as described in the introduction. We refer to frequently reported challenges in getting the patient to talk to the resident and not to the supervisor, and residents’ self-reported unnatural behavior and fear of appearing incompetent. (LaDonna et al., 2017; Rietmeijer et al., 2018; Rietmeijer et al. 2021). Taking this one step further, if we agree that some participation by the supervisor is needed, we should perhaps acknowledge that DO situations do not resemble unobserved resident-patient interactions, but rather that they are unique situations with their own dynamics and preconditions for valuable interactions between all parties. We therefore suggest a new term: Participative direct observation (PDO). ‘Participative’ suggests that some participation by the observing supervisor is involved. Its extent must be negotiated within the triad of patient, resident and supervisor, before, throughout and/or after the encounter, acknowledging the needs of each party. This recommendation may seem difficult to implement in busy clinical practice. However, consciousness of the concept of PDO and its dynamics, and bringing these dynamics into the learning conversation, and occasionally into the consultation, may help all participants.

### A changed concept of direct observation as an educational tool

From an educational perspective, the concept of PDO sheds new light on a persistent challenge concerning the usefulness of DO for workplace learning. From previous research, we know that DO can be highly valuable for teaching and learning, especially when it is regularly planned and bi-directional. However, the threat of assessment often provokes residents’ performance goal orientation and may hinder learning (Rietmeijer et al., 2018, Rietmeijer et al. 2021; LaDonna et al., 2017; Teunissen et al., 2009). This is problematic in itself, and now even more so because we may have to question the appropriateness of DO as an assessment tool, if we agree that the supervisor is not the fly on the wall that sees the resident working with the patient in the same way as when not being observed. Awareness of the concept of PDO and its perhaps questionable value for assessment could help supervisors downplay their role of the silent assessor and, as the clinical teacher, adjust their degree of participation to the needs of both the patient and the resident. This, in turn, could perhaps help temper residents’ performance goal orientation for the benefit of their learning goal orientation (Teunissen et al., 2009). In addition to this, mutual expectations concerning the resident’s individual autonomy could perhaps sometimes make way for explicit mutual intentions to use DO situations for learning together (Rietmeijer & Teunissen, 2019).

## Implications for practice

Our results underline the importance of patients’ informed consent to participate in DO situations. Also, our results suggest that patients should be well informed about—or, even better, engaged in—the intended role division, which may change during the consultation. The patient’s need for contact with the supervisor should be openly addressed and facilitated, rather than ignored or seen as uncalled for. Supervisors should be aware that patients may need signs of their approval of the resident’s approach. And, concluding, residents and supervisors should ensure that DO takes place in a calm and friendly atmosphere. Although these recommendations stem from research in GP training in only one Dutch GP training center, we suggest that they may apply to GP training in general, and perhaps also to other HPE settings.

Adjusting the fly-on-the-wall approach to what the patient, the resident and the supervisor need will probably help make DO feel more natural for patients, and perhaps for residents and supervisors too. Acknowledging DO’s shortcomings for assessment can help both supervisors and residents to use DO more deliberately for teaching and learning. Informed by previous research, we expect that this use of DO will benefit from regularly planning DO sessions and making them bi-directional, meaning that the supervisor and the resident alternately take the role of the doctor and the observer (Young et al. 2020; Voyer et al. (2016) ; Rietmeijer et al., 2018, Rietmeijer et al. 2021). This approach is in line with principles of collaborative learning in which residents and supervisors are both on a learning continuum (Gibson et al., 2019; Rietmeijer & Teunissen, 2019).

## Implications for future research

Our phenomenological approach has resulted in a description of essential elements of experiencing DO situations as a patient. Our findings made us question a core aspect of the current concept of DO in residency, i.e. the role of the supervisor in the DO situation. A similar, phenomenological, investigation of residents’ and supervisors’ experiences with DO could provide other insights that may help construe a concept of DO that fundamentally fits all parties’ needs.

A next step could then be to investigate how to implement this new concept, and to explore the extent to which this would truly enhance both patient-centered care and residents’ learning goal orientation.

Reframing DO situations as primarily learning events and acknowledging the participation of the supervisor brings to light some other perspectives. For instance, it could be worthwhile to investigate the potential of PDO as regularly planned, preferably bi-directional, PDO sessions for supervisors’ continuous professional development.

## Limitations

One can question the feasibility of accurately capturing the experiences and meanings of experiences rather than opinions of it. “Whilst phenomenology as philosophy is associated with introspection, allowing the philosopher to explore his or her experiences through ‘phenomenological meditation’, phenomenology as a research approach relies on the accounts of participants and the experiences of researchers” (Tuffour, 2017). We were aware of this from the start of this project and have therefore followed the methodological steps as described in detail. As indicated, however, how to apply phenomenology to research in HPE is subject to lively debate.

A second limitation is that, for in-depth interviews, the interviews were relatively short. We have identified three reasons for this, the first being that some patients found it very hard to slow down and reflect on what they had just experienced. As a second reason, in the interviews, we focused mostly on the concrete experience of the consultation patients had just had, which was a limited topic. Thirdly, to increase the acceptability for patients of the request to take part, they were recruited with the message that the interview following their consultation would not take too much of their time. It is possible that longer interviews, perhaps with video-stimulated recall, could deliver more information, leading perhaps to more essential elements of the experience of being the patient in a DO situation. On the other hand, we did not stop interviews (except one) because of the time, patients shared their experiences freely and the interview ended when they indicated they had shared their experiences sufficiently.

As another limitation, this study was performed in the context of one GP training center in the Netherlands. Familiarity of patients with their general practitioner (i.e. the supervisor) appeared to be an important feature of this context, explaining one of our major findings. We suspect that in hospital contexts, where patients may know the resident better than the supervisor, or may not know either of them, this element of patients’ experiences will be different.

Lastly, all patients that we interviewed were, coincidentally, rather satisfied with the DO situation that they had just been part of. Dissatisfied patients could well raise new topics of interest. Also, one may question how freely patients will speak about their GP and the resident they just visited when interviewed by a stranger in their doctor’s office.

## Conclusions

Our phenomenological investigation of what it is like to be the patient in a DO situation revealed several essential elements of this experience. As one essence, patients experienced the presence of the supervisor as the presence of a second doctor, a senior doctor, and often, their familiar GP. This led to their need to involve the supervisor, to some extent, in the consultation. Patients were willing to address the resident but sought moments of contact with and some participation by the supervisor. These results highlight the potential side-effects of the fly-on-the-wall approach during DO, regarding patient wellbeing. Also, if we agree that supervisors always to some extent participate in the situation that they are observing, we may have to reconsider the value of DO for assessment, and perhaps primarily deploy DO for learning purposes. We therefore propose the concept of *participative direct observation* (PDO) in workplace learning, which also acknowledges the observer’s role as participant. Embracing this concept may benefit both patients’ wellbeing and residents’ learning.
